# Incorporating work experience of medical staff into patient safety climate management: a multi-group analysis

**DOI:** 10.1186/s12913-018-3747-9

**Published:** 2018-12-03

**Authors:** Seung-Hwan Kim, Shao-Jen Weng

**Affiliations:** 1 Department of Business Administration, Ajou University, Suwon, 443-749 South Korea; 2 Executive Master Program for Health Administration, Tunghai University, Taichung City, Taiwan 40704; 3 Department of Industrial Engineering and Enterprise Information, Tunghai University, Taichung City, Taiwan 40704

**Keywords:** Work experience, Management leadership, Safety climate, Moderation effect

## Abstract

**Background:**

This study aims to provide insights on how to incorporate the work experience of medical staff into safety climate management based on the relationships among several safety-related constructs such as teamwork climate, working condition, and job satisfaction.

**Methods:**

A cross-sectional study was conducted in a regional hospital in Taichung City, Taiwan using a Safety Attitude Questionnaire (SAQ)-based questionnaire. The relationships among the constructs were modeled by a structural equation model, and a multi-group analysis was performed. Among the employees participating in the survey, only physicians and nurses were considered in the analysis, accounting for 1596 out of 2277 responses. The key measures were the difference between the unconstrained and fully constrained structural models, the statistically different coefficients, and their strengths across the high and low-experience groups.

**Results:**

Our multi-group analysis showed that the effects of management leadership on job satisfaction and of teamwork climate on safety climate were statistically stronger for low-experience medical staff, whereas the effect of working conditions on safety climate was statistically stronger for high-experience medical staff.

**Conclusions:**

The findings demonstrate how to incorporate the work experience of medical staff into safety climate management. In summary, by focusing on different safety constructs for the less and more experienced staff—job satisfaction and teamwork climate for the less experienced, working conditions for the more experienced—management may be able to improve the organizational safety climate. Our suggestions in this study can be leveraged, should management implement the initiatives and action plans for safety climate improvement.

## Background

Safety culture is a set of attitudes and values that the members of an organization adhere to regarding safety [1], whereas safety climate has been viewed as a snapshot of safety culture within an organization [1, 2]. Though the culture of an organization is difficult to measure, climate can be measured through quantitative methods; as such, safety climate has been suggested as an effective indicator of the overall safety culture of an organization [3–5]. There have been many studies that have linked safety performance with safety climate [6–11], mostly showing that better safety measures were reported in health care organizations with better safety climate.

One of the organizational factors that affects safety climate is work experience. As previously mentioned, safety climate measures the attitudes and values of employees towards safety, and it has been suggested in human resource management literature that work experience influences work-related attitudes, values, and the performance of employees [12, 13]. Also, it is well known that doctors and nurses have a strong hierarchical culture based on experience and seniority [14, 15]. In safety climate literature, work experience has been demonstrated to have significant associations with attitude, performance, and a climate of organizational safety in a number of studies [16–20]. Notably, Mark et al. [17] shows that work experiences of nurses affects the safety climate of a hospital more than overall nursing education.

Another critical factor that influences safety climate is management leadership [7, 21]. It shows how well managers or supervisors lead and support the medical staff in terms of commitment to safety [22–26]. The impact of management leadership on safety culture or climate has been well documented in the literature of High Reliability Theory (HRT) and the discipline of human resource management [27–32]. HRT investigates how High Reliability Organizations (HROs)—organizations requiring near error-free operations, such as in construction, aviation, and the nuclear power industries—manage to maintain such high reliability in their operations [33]. Numerous safety related studies indicate that the role of management leadership is critical for achieving high levels of safety within organizations [2, 27–32, 34]. In particular, Flin et al. [2] shows that how management leads and commits to safety is the most consistent factor that shapes the safety climate of a workplace.

The aim of this study is to investigate how management should approach the task of managing safety climate of medical staff differently based on the work experience of the staff. There are several measures that can represent work experience of an employee, and in our study the length of time each medical staff has worked in the current department of a hospital was used. Particularly we focus on how work experiences of nurses and doctors moderate the relationships among the construct of management leadership, other safety-related constructs, and the safety climate of a health care organization.

### Research hypothesis

There were two major components to our research hypothesis: 1) the mechanism of how management leadership affects safety climate through other safety-related constructs; 2) how the work experience of doctors and nurses affects the mechanism. For the first component, initially the research model of our previous work in Weng et al. [21] was extended by incorporating the department experience of medical staff as the moderators of all paths in the structural model. Based on previous studies, our model showed that the safety-related constructs of teamwork climate, working conditions, and job satisfaction fully mediated the effect of management leadership on the safety climate of a medical staff [17].

**Fig. 1:**
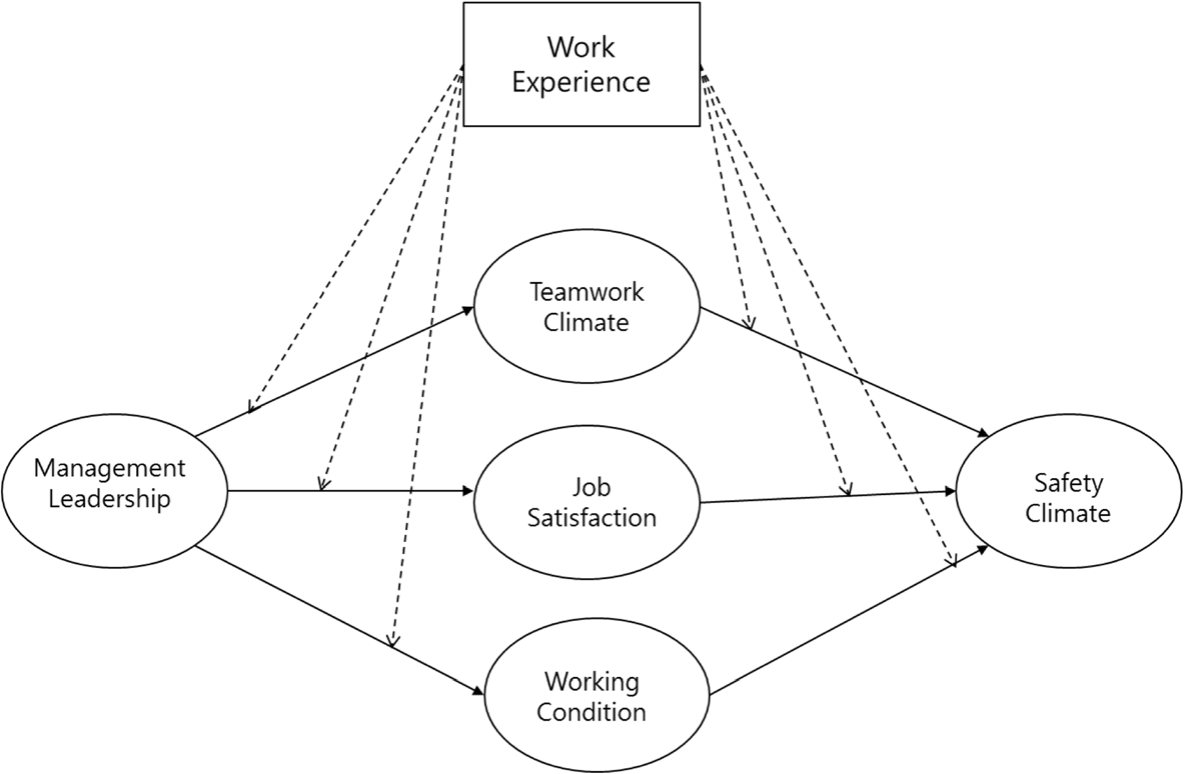
Hypothesized model in the study

For the second component, as mentioned in the background above, it has been shown that work experience is associated with the safety climate of an organization [15–20]. In addition, a number of studies have suggested that the level of work experience may moderate relationships among the perceptions and attitudes for work-related factors of the employees [35–37]. They indicate that inexperienced employees and those with more experience may show different levels of sensitivity to those relationships among the related factors. The safety-related factors considered in this paper—management leadership, teamwork climate, job satisfaction, and working conditions—have been linked with work experience in the literature. First, Vecchio and Boatwright [38] reported that employees with longer job tenure and ones with shorter tenure responded differently regarding their preferences toward management leadership style. Flin et al. [39] and de Wet Carl et al. [40] showed that significant differences were found in issues related to leadership based on the level of experience. Also, Flin et al. [39], Thomas et al. [41], Kim et al. [42], and de Wet Carl et al. [40] indicated that the perception of the teamwork climate of employees is related to their work experience. For job satisfaction, Spector [43], Mwamwenda [44], and Traut et al. [45] suggest that the degree to which people are satisfied with their job may differ based on their length of employment. In terms of working conditions, Cox and Cheyne [46], and Mearns et al. [47] point out that the degree of influence people get from their working environment may vary with respect to their level of experience. Based on the aforementioned literature, this study examined the work experience of medical staff as a moderator in the relationships among management leadership, teamwork climate, job satisfaction, working conditions, and safety climate, and hypothesized that the strength of those relationships would not be the same amongst those with different levels of work experience. By combining the first and the second components, Fig. 1 presents the hypothesized model in this study.

## Methods

### Setting and sample

Our research was performed based on a cross-sectional study conducted at Taichung Veterans General Hospital in Taichung City, Taiwan. This hospital is a 1500-bed hospital with around 3000 employees. At full capacity, about 6000 outpatients are treated every day, with about 130 inpatients and 180 patients in the emergency room. Physicians, nurses, and other employees in the hospital were required to participate in the survey, but not all questions in the survey were suitable for all staff members. Therefore, we only considered the responses from physicians and nurses (1596 responses out of the total 2277).

### Ethical approval

Ethical approval for this study was obtained from the Institutional Review Board of Taichung Veterans General Hospital in Taiwan (IRB TCVGH No: CW15250A).

### Measures and instruments

We adapted questions from the self-administered Safety Attitude Questionnaire (SAQ) [48] and the new Chinese version of SAQ [49], which have been validated and widely used in the health care industry [50, 51]. There are five safety-related constructs in the questionnaire—safety climate, management leadership, teamwork climate, job satisfaction, and working conditions. To measure management leadership, we selected questions from the “hospital management support” scale developed by the new Chinese version of SAQ [49] and the dimension of “perception of management” in the original SAQ. The other constructs were measured by adapting questions from the original SAQ [48]. The construct ‘stress recognition’ in the original SAQ was excluded from our study based on Taylor and Pandian [51], Sexton et al. [48], and Lee et al. [52], which showed stress recognition does not fit into the overall safety climate construct in SAQ. Safety climate reflects the strength of the perception of a strong and proactive organizational commitment to safety [48]. Management leadership assesses the perception of medical staff on how well the managers lead and support the medical staff in terms of commitment to safety [48]. Teamwork climate is the perceived quality of collaboration among personnel [48]. Job satisfaction shows the degree to which employees feel positive about their work experience [48]. Working conditions are defined as the perceived quality of the work environment, such as staff training and information support [48]. We chose the tenure in the department, not in the hospital to measure work experience of the medical staff. The reason the tenure in the hospital was not chosen was that the learning status of nurses can be affected by ‘job rotation’, which transfers nurses from one department to another. Consequently, we assumed that an employee with long hospital tenure do not always show higher skill and comfort level on their job. By choosing the department tenure, we assumed in our study that the length of tenure has a positive linear relationship with the skill and comfort level. The participating physicians and nurses were categorized into two groups based on their ‘department work experience’ which has seven levels in the questionnaire. In our experiment, the first three levels (Under 6 months, 6–11 months, 1–2 years) were defined as ‘low’ and the last three levels (5–10 years, 11–20 years, Above 21 years) as ‘high’. The median level (3–4 years) was excluded due to its ambiguity in terms of the level of experience, which may hinder our objective in differentiating the effect of high and low work experience. The respondents answered each question based on a five-point Likert scale ranging from ‘strongly agree’ to ‘strongly disagree’, or frequencies delineated as ‘never’, ‘rarely’, ‘sometimes’, ‘most of the time’, and ‘always’.

### Data analysis

To perform the analysis, the research model in Fig. 1 was formulated into a structural equation model (SEM) with AMOS ver. 21.0. SEM provides an advanced platform for analyzing mediated or moderated relationships among not only variables but also constructs that encompass variables. First, we verified the full mediation of teamwork climate, job satisfaction, and working conditions on the effect of management leadership to safety climate by analyzing the structural equation model. Next, based on Anderson and Gerbing [53], the two-step approach was employed where the measurement model is examined first with a confirmatory factor analysis (CFA). CFA allows researchers to test their hypotheses that certain relationships between observed variables and their underlying latent constructs exist, when the structure of observed variables and their constructs are predetermined based on existing studies. Then it is followed by a multi-group analysis of the structural model for two different levels of the department experience of the medical staff in the hospital.

## Results

Among the physicians and nurses in the hospital (2205), 1596 participants gave valid responses, which resulted in a response rate of 72.38%.The analysis of measurement model and structural model was then performed based on the aforementioned responses from the survey. The overall demographic information of the participants is summarized in Table 1.

**Table 1:** The demographic information of the participants

Category	Frequency	Percentage
Gender		
Male	202	12.65
Female	1394	87.34
Age		
Less than 30 years old	696	43.60
31–40 years old	446	27.94
41–50 years old	354	22.18
51–60 years old	92	5.76
61 years old and above	8	0.50
Types of job		
Physicians	244	15.28
Nurses	1352	84.71
Working experience in hospital		
Under 6 months	90	5.63
6–11 months	59	3.69
1–2 years	306	19.17
3–4 years	214	13.40
5–10 years	343	21.49
11–20 years	353	22.11
Above 21 years	231	14.47
Working experience in department		
Under 6 months	141	8.83
6–11 months	90	5.63
1–2 years	364	22.8
3–4 years	284	17.79
5–10 years	382	23.93
11–20 years	235	14.72
Above 21 years	100	6.26
Education		
Under junior	1	0.06
High school	5	0.31
College	1404	87.96
Master’s degree or above	186	11.65

Table 2 presented descriptive statistics for the constructs considered in the study. It showed that all the observed variables had absolute values of skewness less than 2 and absolute values of kurtosis less than 7, indicating sufficient univariate normality.

**Table 2:** Descriptive Statistics

	Range	Mean	Std. Deviation	Skewness	Kurtosis
High Work Experience (*N* = 717)					
Teamwork Climate	3.50	4.0600	.77988	−.769	.030
Safety Climate	3.43	4.0650	.71056	−.634	−.240
Job Satisfaction	4.00	4.0271	.88260	−.726	−.060
Management Leadership	3.33	3.9370	.74812	−.343	−.749
Working Conditions	3.25	4.0830	.79206	−.494	−.650
Low Work Experience (*N* = 595)					
Teamwork Climate	4.00	4.1303	.70690	−.810	.515
Safety Climate	4.00	4.0953	.66181	−.859	1.097
Job Satisfaction	4.00	3.9650	.84661	−.584	−.059
Management Leadership	4.00	3.9616	.69347	−.524	.436
Working Conditions	4.00	4.0731	.77938	−.568	−.071

### Measurement model

A confirmatory factor analysis (CFA) was carried out for the total sample of 1312 responses (High: 717, Low: 595) to validate the latent constructs measured with the original 29 items in the overall measurement model. Five items with low associated factor loadings were then removed (two from ‘Teamwork Climate’ construct, three from ‘Safety Climate’ construct). The resulting overall measurement model with 24 measures showed a good fit with χ2 of 874.809 (df = 212, *p* < 0.001); goodness-of fit index (GFI) = 0.927; adjusted goodness-of fit index (AGFI) = 0.895; Tucker–Lewis index (TLI) = 0.949; comparative fit index (CFI) = 0.961; and root mean square error of approximation (RMSEA) = 0.049.

We then tested measurement invariance based on Byrne [54] and Yu [55], where the measurement invariance of factor loadings was tested before assessing the invariance of individual path coefficients for analyzing moderation effects. As mentioned above, the overall measurement model with all model parameters to be estimated freely in both groups showed a χ2 value of 874.809 (df = 212, *p* < 0.001). The factor loadings were then constrained to be equal across the groups, resulting in a χ2 value of 893.036 (df = 224, p < 0.001). The difference between these two test statistics (χ2 with df = 12) was 18.23, which was not significant (*p* = 0.109), implying that the measures showed full metric invariance. Since we verified measurement invariance of factor loadings, a multi-group CFA was conducted to check the construct validity of the measurement model. Table 3 presented the outcome of a multi-group CFA. All the items were loaded significantly on their corresponding constructs (*t* > 1.96) with their factor loadings all above 0.6. The scale reliabilities of all constructs, which were measured by Cronbach’s alpha, exceeded 0.7 and the composite reliability (CR) values of the constructs were all above 0.8. Also, the average variance extracted (AVE) values of all constructs were over 0.5. These results all exceeded customary acceptable levels [56, 57], indicating good construct validity of the measurement model.

**Table 3:** Multi-group CFA results

Construct	Item	High Experience					Low Experience				
		α	CR	AVE	Factor Loading	*t*-value	α	CR	AVE	Factor Loading	*t*-value
Management Leadership	1	0.803	0.827	0.617	0.822	–	0.787	0.824	0.612	0.784	–
	2				0.855	35.163				0.861	35.163
	3				0.652	24.563				0.640	24.563
Teamwork Climate	1	0.828	0.807	0.582	0.739	–	0.779	0.786	0.551	0.702	–
	2				0.818	27.579				0.795	27.579
	3				0.810	26.493				0.719	26.493
Job Satisfaction	1	0.927	0.936	0.786	0.756	–	0.923	0.933	0.778	0.742	–
	2				0.917	31.259				0.908	31.259
	3				0.933	36.424				0.942	36.424
	4				0.940	36.238				0.921	36.238
Working Conditions	1	0.828	0.854	0.662	0.845	–	0.827	0.859	0.670	0.876	–
	2				0.764	27.870				0.808	27.870
	3				0.859	31.148				0.844	31.148
Safety Climate	1	0.872	0.886	0.659	0.827	–	0.840	0.873	0.633	0.760	–
	2				0.792	30.908				0.784	30.908
	3				0.814	31.921				0.801	31.921
	4				0.845	29.360				0.866	29.360

*a* Cronbach alpha, *CR* Composite Reliability, *AVE* Average Variance Extracted

### Structural model

To test the moderating effect of work experience hypothesized in Fig. 1, a two-group analysis was performed on the structural model. First, the unconstrained model with all path coefficients to be estimated freely in both groups results in a good fit—χ2 of 872.193 (df = 214, *p* < 0.001); GFI = 0.928; AGFI = 0.897; TLI = 0.950; CFI = 0.961; and RMSEA = 0.048. The unconstrained model was then compared with the fully constrained model, where all the paths were constrained to be equal across the two groups. The difference between the test statistics of the two models (χ2 with df = 6) was 25.143, which was significant (p < 0.001). Therefore, there was evidence that the structural coefficients did differ across the high and low-experience groups. Table 4 presented the results of structural models for the two experience groups.

**Table 4:** Results of structural models

Path	High Experience			Low Experience		
	Unstd. Est. *t-*value		*P*	Unstd. Est. *t-*value		*P*
ML -- > TEAMWK	0.714	16.315	< 0.001	0.790	14.460	< 0.001
ML -- > JOBSF	0.662	18.472	< 0.001	0.799	15.578	< 0.001
ML -- > WORKCN	0.983	24.730	< 0.001	0.968	17.623	< 0.001
TEAMWK -- > SAFECLMT	0.465	10.109	< 0.001	0.739	9.513	< 0.001
JOBSF -- > SAFECLMT	0.098	2.105	0.035	0.131	2.933	0.003
WORKCN -- > SAFECLMT	0.375	8.200	< 0.001	0.091	1.834	0.067

*Unstd*. *Est*. Unstandardized Structural Coefficient, *P p*-value, *ML* Management Leadership, *SAFECLMT* Safety Climate, *TEAMWK* Teamwork Climate, *WORKCN* Working Conditions, *JOBSF* Job Satisfaction

All structural coefficients in both groups were statistically significant, with the exception of the path from working conditions to safety climate—the effect was significant in the high-experience group, but not significant in the low-experience group. In two paths, the effects were stronger in the high-experience group (management leadership➔ working conditions, working conditions➔safety climate) and for the other four paths, the effects were stronger in the low-experience group (management leadership➔job satisfaction, management leadership➔teamwork climate, teamwork climate➔safety climate, job satisfaction➔safety climate). To identify which particular effects differed statistically between the two groups, two models were compared at a time—the unconstrained model, and a model in which a particular path coefficient of interest was constrained. The statistical difference between the two models was then examined for each path. The outcome of the pairwise comparisons was presented in Table 5.

**Table 5:** Path constrained model comparisons

Constrained Path	X^2^	df	Adf	A%^2^	P
None (Unconstrained model)	872.193	214	–	–	–
All (Fully constrained model)	897.336	220	6	25.143	< 0.001
ML -- > TEAMWK	873.335	215	1	1.143	0.285
ML -- > JOBSF	877.097	215	1	4.904	0.027
ML -- > WORKCN	872.236	215	1	0.044	0.834
TEAMWK -- > SAFECLMT	880.918	215	1	8.726	0.003
JOBSF -- > SAFECLMT	872.439	215	1	0.247	0.62
WORKCN -- > SAFECLMT	887.947	215	1	15.754	< 0.001

*ML* Management Leadership, *SAFECLMT* Safety Climate, *TEAMWK* Teamwork Climate, *JOBSF* Job Satisfaction, *WORKCN* Working Conditions

The study showed that the effects of management leadership on job satisfaction, teamwork climate on safety climate, and working conditions on safety climate were found to be statistically different across the high and low-experience groups. Therefore, we inferred that the effects of management leadership on job satisfaction and teamwork climate on safety climate were stronger for low-experience medical staff, whereas the effect of working conditions on safety climate was stronger for high-experience medical staff.

## Discussion

In this paper we investigate how management may incorporate the work experience of medical staff in managing the safety climate of a health care organization. A multi-group analysis was conducted for the medical staff of a hospital with two different levels of department work experience. Our results showed that there was evidence of the structural coefficients differing across the high and low-experience medical staff. By performing path-constrained model comparisons, the coefficients that were statistically different in those two groups were identified.

First, the multi-group analysis revealed that the effect of management leadership on job satisfaction was significantly stronger in the low-experience group. It has been reported that job satisfaction of employees is affected by how managers lead them [58–62] and the degree of job satisfaction generally increases as the work experience and tenure of employees increase [43–45]. Even though there can be multiple factors that can contribute to the increase of job satisfaction with the increase of work experience, one of the factors is tolerance for the influence of authority [43]—as people accumulate more experience in an organization, they become more capable of dealing with the influence of managers with higher authority. Therefore, one possible explanation for our result is that the medical staff with low experience may be more sensitive (or less tolerant) to the influence of management on their job satisfaction. For this finding, the implication of how to incorporate work experience into safety climate management is that by showing more care and commitment particularly to the less experienced staff, management may be able to improve the safety climate of an organization indirectly by improving the job satisfaction of the less experienced, since job satisfaction mediates the effect of management leadership on safety climate.

Second, the multi-group analysis showed that the effect of teamwork climate on safety climate was statistically stronger in the low-experience group. The teamwork climate construct in our measurement reflects the comfort level of each individual when working with other team members, which is closely related to ‘socialization’ in an organization [63]. Socialization in an organization is the process in which newcomers become ‘insiders’ of an organization [64]. As people in an organization gain more experience and interact more extensively with others, their socialization process advances and they become more like ‘insiders’ who are comfortable in working with other members and adapting to the norms of the organization [65]. Therefore, our result may be attributed to their low level of socialization—since low-experienced members are not comfortable enough in working with other team members, their perception of safety climate may be affected more by their collaboration with other team members than the perception of the highly experienced. For the aforementioned finding, the implication on how to incorporate work experience into safety climate management is that the management may have to put more effort into facilitating the socialization process of less experienced staff (i.e. aiding the less experienced staff by implementing mentoring programs) in order to affect their perception of teamwork climate positively; this can significantly improve their safety climate, since teamwork climate has been reported as a highly critical factor that affects patient safety [52, 66–68].

Furthermore, the analysis demonstrated that the effect of working conditions on safety climate was significantly stronger in the high-experience group. One of the key components that the construct of working conditions in our measurement deals with is about staff training and supervision, especially for new and inexperienced staff members [48]. The reasoning is that working with less-trained, inexperienced personnel can seriously threaten the safety of patients and medical staff. Flin et al. [39] investigated the influence of seniority in terms of work experience in their study of 11 hospitals, and found that the degree to which medical staff is irritated by improperly trained members significantly differed between consultants (more experienced) and non-consultants (less experienced)—the medical stuff with more experience became more irritated than the less experienced. This may be related to the outcome of our results. It is possible that more experienced medical staff is more irritated and threatened by the incompetence of co-workers, which can negatively affect safety climate. The incompetence is mainly caused by the issues of training and supervising new personnel, which is measured by the construct of “working conditions” in our study. Therefore, we may say that working conditions affect the experienced staff more than the less experienced, consequently results in a stronger effect on safety climate. Regarding our discovery mentioned above, the implication on how to incorporate work experience into safety climate management is not only that management must continue to train new personnel and educate medical staff regarding safety issues, but they also need to pay more attention to the complaints of experienced medical staff regarding the members that cause problems, and try to show strong commitment to deal with those personnel in a constructive manner. The management may then be able to positively affect the perception of working conditions for more experienced staff, which can result in improved safety climate.

### Limitations

There are several limitations in our study that can lead to more future works. One concern is the limitation of reflecting different aspects of the safety related constructs. In this study, our measurement tool was based on two SAQ-based questionnaires. Even though SAQ is a validated and widely used tool in the health care industry [50, 51], the safety-related constructs in SAQ can only measure certain aspects of each construct, and therefore our interpretations may be limited to those aspects. For example, the working condition construct in our study mainly describes the issues of training and supervising new medical staff, whereas there may be health care organizations more concerned about other issues of working conditions such as the effectiveness and maintenance of equipment or the overall workforce levels of organizations compared to their demand. For this reason, future research may utilize other measurement tools that address different aspects of safety constructs and extend the investigation of this topic.

Second, future research may further investigate the impact of cultural differences in different regions of the world. How people perceive and behave responding to their management or seniors can vary under different cultures, which in turn may draw different dynamics between management leadership constructs and the other safety constructs. This study was conducted in Taiwan, which may limit our results to the Southeast Asia region. Consequently, we suggest researchers extend this study to various cultures in order to compare and analyze the outcomes.

Third, the perceptions of people regarding certain constructs of organizations can change over time. Since our study was based on cross-sectional data, it would be meaningful to perform a longitudinal study that analyzes how the impact of work experience on the relationships among the safety-related constructs considered in this study change over time so that management may understand trends and construct future plans for organizational safety in the long term.

Lastly, we excluded the medium level of work experience (3–4 years) from the analysis. The reason we excluded the medium level was not theoretical, but rather practical based on our previous experience. Our main objective is to differentiate the effect of work experience on the relationships among the constructs based on two clearly different levels of the moderator (work experience). In many cases when the proportion of the medium level is significant (In our case, about 18%), we have seen that it could water down the effect in a way that it hinders our objective in differentiating the effect of high and low moderator. An investigation of multi-level analysis on the work experience moderation of safety dimensions can be meaningful in the future.

## Conclusions

This study investigated how to incorporate the work experience of medical staff into safety climate management. Our results suggested that management should approach the more experienced and the less experienced differently by focusing on different safety constructs—more on job satisfaction and teamwork climate for the less experienced, and more on working conditions for the more experienced. The findings in this study may be leveraged for efficient utilization of limited resources for safety climate improvement, especially at the planning stage of the initiatives and action plans for the organization.

## Abbreviations

AGFI: Adjusted goodness-of fit index
AVE: Average variance extracted
CFA: Confirmatory factor analysis
CFI: Comparative fit index
CR: Composite reliability
GFI: Goodness-of fit index
HROs: High reliability organizations
HRT: High reliability theory
RMSEA: Root mean square error of approximation
SAQ: Safety attitude questionnaire
SEM: Structural equation modeling
TLI: Tucker–lewis index
